# Deaths and Years of Potential Life Lost From Excessive Alcohol Use — United States, 2011–2015

**DOI:** 10.15585/mmwr.mm6939a6

**Published:** 2020-10-02

**Authors:** Marissa B. Esser, Adam Sherk, Yong Liu, Timothy S. Naimi, Timothy Stockwell, Mandy Stahre, Dafna Kanny, Michael Landen, Richard Saitz, Robert D. Brewer

**Affiliations:** ^1^Division of Population Health, National Center for Chronic Disease Prevention and Health Promotion, CDC; ^2^Canadian Institute for Substance Use Research, University of Victoria, British Columbia, Canada; ^3^Boston Medical Center, Boston, Massachusetts; ^4^Boston University Schools of Medicine and Public Health, Boston, Massachusetts; ^5^Forecasting and Research, State of Washington Office of Financial Management; ^6^New Mexico Department of Health.

Excessive alcohol use is a leading cause of preventable death in the United States (*1*) and costs associated with it, such as those from losses in workplace productivity, health care expenditures, and criminal justice, were $249 billion in 2010 (*2*). CDC used the Alcohol-Related Disease Impact (ARDI) application* to estimate national and state average annual alcohol-attributable deaths and years of potential life lost (YPLL) during 2011–2015, including deaths from one’s own excessive drinking (e.g., liver disease) and from others’ drinking (e.g., passengers killed in alcohol-related motor vehicle crashes). This study found an average of 95,158 alcohol-attributable deaths (261 deaths per day) and 2.8 million YPLL (29 years of life lost per death, on average) in the United States each year. Of all alcohol-attributable deaths, 51,078 (53.7%) were caused by chronic conditions, and 52,921 (55.6%) involved adults aged 35–64 years. Age-adjusted alcohol-attributable deaths per 100,000 population ranged from 20.8 in New York to 53.1 in New Mexico. YPLL per 100,000 population ranged from 631.9 in New York to 1,683.5 in New Mexico. Implementation of effective strategies for preventing excessive drinking, including those recommended by the Community Preventive Services Task Force (e.g., increasing alcohol taxes and regulating the number and concentration of alcohol outlets), could reduce alcohol-attributable deaths and YPLL.^†^

CDC has updated the ARDI application, including the causes of alcohol-attributable death, *International Classification of Diseases, Tenth Revision* codes,^§^ and alcohol-attributable fractions.^¶^ CDC used ARDI to estimate the average number of annual national and state alcohol-attributable deaths and YPLL caused by excessive drinking (i.e., deaths from conditions that are 100% alcohol-attributable, acute conditions that involved binge drinking, and chronic conditions that involved medium or high average daily alcohol consumption). ARDI estimates alcohol-attributable deaths by multiplying the total number of deaths (based on vital statistics) with an underlying cause corresponding to any of the 58 alcohol-related conditions in the ARDI application by its alcohol-attributable fraction. Some conditions (e.g., alcoholic liver cirrhosis) are wholly (100%) attributable to alcohol (alcohol-attributable fraction = 1.0), whereas others are partially attributable (alcohol-attributable fraction <1.0) to alcohol (e.g., breast cancer and hypertension). Deaths are assessed by age group and sex and averaged over a 5-year period. The alcohol-attributable fractions for chronic conditions are generally calculated using relative risks from published meta-analyses and the prevalence of low, medium, and high average daily alcohol consumption among U.S. adults, based on data from the Behavioral Risk Factor Surveillance System.** The prevalence estimates are adjusted to account for underreporting of alcohol use during binge drinking episodes (*3*). Alcohol-attributable fractions for acute causes (e.g., injuries) are generally based on studies that measured the proportion of decedents who had a blood alcohol concentration ≥0.10 g/dL (*4*). Alcohol-attributable fractions for motor vehicle crash deaths are based on the proportion of crash deaths that involved a blood alcohol concentration ≥0.08 g/dL.^††^ For 100% alcohol-attributable conditions, deaths are summed without adjustment.^§§^ YPLL, a commonly used measure of premature death, are calculated by multiplying the age-specific and sex-specific alcohol-attributable deaths by the corresponding reduction in years of life potentially remaining for decedents relative to average life expectancies.^¶¶^ Chronic causes of death are calculated for decedents aged ≥20 years, and acute causes are generally calculated for decedents aged ≥15 years. Deaths involving children that were caused by someone else’s drinking (e.g., deaths caused by a pregnant mother’s drinking and passengers killed in alcohol-related motor vehicle crashes) are also included.

CDC used the data available in ARDI to estimate the average annual national and state alcohol-attributable deaths and YPLL associated with excessive drinking and national estimates of alcohol-attributable deaths and YPLL by cause of death, sex, and age group. National and state alcohol-attributable deaths and YPLL per 100,000 population were calculated by dividing the average annual alcohol-attributable death and YPLL estimates, respectively, by average annual population estimates from the U.S. Census for 2011–2015, and then multiplying by 100,000. The alcohol-attributable death rates were then age-adjusted to the 2000 U.S. population.*** The number of YPLL per alcohol-attributable death was calculated by dividing total YPLL by total alcohol-attributable deaths in the United States and in states.

During 2011–2015 in the United States, an average of 95,158 alcohol-attributable deaths occurred, and 2.8 million years of potential life were lost annually (29.0 YPLL per alcohol-attributable death) (Table 1) (Table 2). Among the 95,158 deaths, 51,078 (53.7%) were caused by chronic conditions and 44,080 (46.3%) by acute conditions. Of the 2.8 million YPLL, 1.1 million (40.0%) were because of chronic conditions, and 1.7 million (60.0%) were because of acute conditions. Overall, 67,943 (71.4%) alcohol-attributable deaths and 2.0 million (71.0%) YPLL involved males. Among all alcohol-attributable deaths, 52,921 (55.6%) involved adults aged 35–64 years, 24,972 (26.2%) involved adults aged ≥65, and 14,819 (15.6%) involved young adults aged 20–34 years (Figure).

**TABLE 1:** Average annual number of deaths and years of potential life lost attributable to excessive alcohol use,* by condition and sex — United States, 2011–2015

Cause	Alcohol-attributable deaths			Years of potential life lost		
	Total^†^	Males no. (%)	Females no. (%)	Total^†^	Males no. (%)	Females no. (%)
**Total^†^**	**95,158**	**67,943 (71.4)**	**27,215 (28.6)**	**2,763,055**	**1,962,436 (71.0)**	**800,619 (29.0)**
**Chronic causes**	51,078	35,583 (69.7)	15,495 (30.3)	1,105,190	752,936 (68.1)	352,253 (31.9)
Alcohol abuse	2,591	1,986 (76.6)	605 (23.4)	66,839	49,129 (73.5)	17,710 (26.5)
Alcohol cardiomyopathy	510	432 (84.7)	78 (15.3)	12,235	10,136 (82.8)	2,099 (17.2)
Alcohol dependence syndrome	4,258	3,269 (76.8)	989 (23.2)	109,911	81,192 (73.9)	28,719 (26.1)
Alcohol polyneuropathy	3	3 (100.0)	0 (—)	54	54 (100.0)	0 (—)
Alcoholic gastritis	33	26 (78.8)	7 (21.2)	890	696 (78.2)	194 (21.8)
Alcoholic liver disease	18,164	12,887 (70.9)	5,277 (29.1)	467,996	313,897 (67.1)	154,099 (32.9)
Alcoholic myopathy	0	0 (—)	0 (—)	0	0 (—)	0 (—)
Alcoholic psychosis	703	549 (78.1)	154 (21.9)	14,129	10,799 (76.4)	3,330 (23.6)
Alcohol-induced acute pancreatitis	278	214 (77.0)	64 (23.0)	8,284	6,247 (75.4)	2,037 (24.6)
Alcohol-induced chronic pancreatitis	52	38 (73.1)	14 (26.9)	1,507	1,046 (69.4)	461 (30.6)
Atrial fibrillation	329	228 (69.3)	100 (30.4)	2,943	2,084 (70.8)	860 (29.2)
Cancer, breast (females only)	584	NA	584 (NA)	11,203	NA	11,203 (NA)
Cancer, colorectal	996	898 (90.2)	98 (9.8)	15,540	14,016 (90.2)	1,524 (9.8)
Cancer, esophageal^§^	494	430 (87.0)	64 (13.0)	8,038	7,007 (87.2)	1,031 (12.8)
Cancer, laryngeal	248	233 (94.0)	15 (6.0)	4,002	3,737 (93.4)	265 (6.6)
Cancer, liver	1,609	1,545 (96.0)	64 (4.0)	28,191	27,129 (96.2)	1,061 (3.8)
Cancer, oral cavity and pharyngeal	909	830 (91.3)	79 (8.7)	16,034	14,715 (91.8)	1,319 (8.2)
Cancer, pancreatic^¶^	186	151 (81.2)	35 (18.8)	2,827	2,301 (81.4)	526 (18.6)
Cancer, prostate (males only)	188	188 (NA)	NA	1,952	1,952 (NA)	NA
Cancer, stomach^¶^	58	56 (96.6)	3 (5.2)	943	897 (95.1)	46 (4.9)
Chronic hepatitis	2	2 (100.0)	0 (0.0)	42	36 (85.7)	6 (14.3)
Coronary heart disease	3,537	2,971 (84.0)	567 (16.0)	46,698	40,183 (86.0)	6,515 (14.0)
Degeneration of nervous system attributable to alcohol	145	118 (81.4)	27 (18.6)	2,617	2,030 (77.6)	587 (22.4)
Esophageal varices	112	77 (68.8)	34 (30.4)	2,414	1,711 (70.9)	703 (29.1)
Fetal alcohol syndrome	4	2 (50.0)	2 (50.0)	212	122 (57.5)	90 (42.5)
Fetus and newborn affected by maternal use of alcohol	1	1 (100.0)	0 (0.0)	76	76 (100.0)	0 (—)
Gallbladder disease	0	0 (—)	0 (—)	0	0 (—)	0 (—)
Gastroesophageal hemorrhage	31	20 (64.5)	10 (32.3)	517	359 (69.4)	157 (30.4)
Hypertension	3,584	1,638 (45.7)	1,946 (54.3)	50,016	26,021 (52.0)	23,994 (48.0)
Infant death, low birthweight**	2	1 (50.0)	1 (50.0)	133	69 (51.9)	65 (48.9)
Infant death, preterm birth**	44	24 (54.5)	19 (43.2)	3,410	1,845 (54.1)	1,565 (45.9)
Infant death, small for gestational age**	0	0 (—)	0 (—)	13	5 (38.5)	7 (53.8)
Liver cirrhosis, unspecified	9,801	5,696 (58.1)	4,105 (41.9)	197,875	114,580 (57.9)	83,295 (42.1)
Pancreatitis, acute	0	0 (—)	0 (—)	0	0 (—)	0 (—)
Pancreatitis, chronic	15	12 (80.0)	3 (20.0)	317	252 (79.5)	65 (20.5)
Pneumonia^††^	133	105 (78.9)	29 (21.8)	3,714	2,839 (76.4)	875 (23.6)
Portal hypertension	61	34 (55.7)	26 (42.6)	1,267	729 (57.5)	538 (42.5)
Stroke, hemorrhagic	938	565 (60.2)	374 (39.9)	14,497	8,856 (61.1)	5,641 (38.9)
Stroke, ischemic	342	243 (71.1)	100 (29.2)	3,867	2,837 (73.4)	1,030 (26.6)
Unprovoked seizures, epilepsy, or seizure disorder	134	112 (83.6)	22 (16.4)	3,987	3,352 (84.1	635 (15.9)
**Acute causes**	44,080	32,360 (73.4)	11,720 (26.6)	1,657,865	1,209,500 (73.0)	448,365 (27.0)
Air-space transport	75	64 (85.3)	11 (14.7)	2,268	1,867 (82.3)	401 (17.7)
Alcohol poisoning	2,288	1,735 (75.8)	553 (24.2)	76,224	56,511 (74.1)	19,713 (25.9)
Aspiration	255	141 (55.3)	114 (44.7)	4,765	2,695 (56.6)	2,070 (43.4)
Child maltreatment^§§^	148	87 (58.8)	61 (41.2)	11,000	6,294 (57.2)	4,706 (42.8)
Drowning	1,043	820 (78.6)	223 (21.4)	35,969	28,803 (80.1)	7,167 (19.9)
Fall injuries^¶¶^	2,015	1,427 (70.8)	588 (29.2)	53,954	38,009 (70.4)	15,945 (29.6)
Fire injuries	1,066	640 (60.0)	426 (40.0)	25,550	15,145 (59.3)	10,405 (40.7)
Firearm injuries	129	109 (84.5)	20 (15.5)	4,947	4,124 (83.4)	823 (16.6)
Homicide	7,334	5,899 (80.4)	1,436 (19.6)	318,006	258,572 (81.3)	59,434 (18.7)
Hypothermia	296	194 (65.5)	102 (34.5)	6,199	4,354 (70.2)	1,845 (29.8)
Motor-vehicle nontraffic crashes	190	144 (75.8)	47 (24.7)	5,588	4,249 (76.0)	1,339 (24.0)
Motor-vehicle traffic crashes***	7,092	5,522 (77.9)	1,570 (22.1)	323,610	245,447 (75.8)	78,163 (24.2)
Occupational and machine injuries	126	117 (92.9)	9 (7.1)	3,294	3,060 (92.9)	234 (7.1)
Other road vehicle crashes	170	137 (80.6)	33 (19.4)	5,632	4,473 (79.4)	1,159 (20.6)
Poisoning (not alcohol)	11,839	7,524 (63.6)	4,315 (36.4)	444,235	280,270 (63.1)	163,965 (36.9)
Suicide	9,899	7,711 (77.9)	2,189 (22.1)	332,791	252,674 (75.9)	80,117 (24.1)
Suicide by and exposure to alcohol	38	24 (63.2)	14 (36.8)	1,267	764 (60.3)	503 (39.7)
Water transport	75	65 (86.7)	9 (12.0)	2,566	2,189 (85.3)	377 (14.7)

**Abbreviation:** NA = not applicable.

* In the Alcohol-Related Disease Impact application (https://www.cdc.gov/ARDI), deaths attributable to excessive alcohol use include deaths from 1) conditions that are 100% alcohol-attributable, 2) deaths caused by acute conditions that involved binge drinking, and 3) deaths caused by chronic conditions that involved medium (>1 to ≤2 drinks of alcohol [women] or >2 to ≤4 drinks [men]) or high (>2 drinks of alcohol [women] or >4 drinks [men]) levels of average daily alcohol consumption.

^†^ Numbers might not sum to totals, and row percentages might not sum to 100% because of rounding.

^§^ Deaths calculated for the proportion of esophageal cancer deaths caused by squamous cell carcinoma only, based on the Surveillance, Epidemiology, and End Results data in 18 states (SEER18). https://seer.cancer.gov/.

^¶^ Deaths among those consuming high average daily levels of alcohol only.

** Alcohol consumption prevalence estimates calculated among women aged 18–44 years only.

^††^ Deaths among persons aged 20–64 years only because of the high number of deaths from pneumonia among persons aged ≥65 years that are not alcohol-related and the lack of relative risks that differ by age.

^§§^ Deaths among persons aged 0–14 years.

^¶¶^ Deaths among persons aged 15–69 years only because of the high number of deaths from falls among persons aged ≥70 years that are not alcohol-attributable and the lack of alcohol-attributable fractions that differ by age.

*** Deaths among persons of all ages. A blood alcohol concentration level of ≥0.08 g/dL is used for defining alcohol attribution for this condition.

**TABLE 2:** Annual average number of deaths and years of potential life lost from excessive alcohol use,* by state — United States, 2011–2015

Location	Alcohol-attributable deaths	Age-adjusted alcohol-attributable deaths per 100,000-population	Years of potential life lost	Years of potential life lost per 100,000-population	Years of potential life lost per alcohol-attributable death
**U.S. total**	**95,158**	**28.0**	**2,763,055**	**873.0**	**29.0**
Alabama	1,504	29.2	46,347	959.4	30.8
Alaska	297	40.0^†^	9,794	1,335.5	33.0
Arizona	2,629	37.5	76,039	1,144.8	28.9
Arkansas	923	29.4	27,699	936.3	30.0
California	11,026	27.5	308,831	803.8	28.0
Colorado	1,821	32.7	54,564	1,033.6	30.0
Connecticut	913	23.2	26,366	733.8	28.9
Delaware	278	27.6^†^	8,445	911.5	30.4
District of Columbia	219	33.0^†^	6,440	994.6	29.4
Florida	6,903	30.4	188,713	960.6	27.3
Georgia	2,637	25.6	79,017	789.6	30.0
Hawaii	349	22.3^†^	9,482	674.3	27.2
Idaho	493	29.5	14,099	872.2	28.6
Illinois	3,391	24.8	100,018	776.9	29.5
Indiana	1,946	28.1	58,407	889.2	30.0
Iowa	841	24.8	22,266	719.8	26.5
Kansas	764	25.2	22,725	785.5	29.7
Kentucky	1,552	33.0	46,452	1,056.4	29.9
Louisiana	1,591	33.0	50,180	1,084.9	31.5
Maine	427	27.2^†^	11,375	855.8	26.6
Maryland	1,505	23.8	46,185	778.8	30.7
Massachusetts	1,744	23.6	49,020	731.0	28.1
Michigan	3,205	29.7	92,753	936.8	28.9
Minnesota	1,343	22.9	37,011	683.0	27.6
Mississippi	954	30.7	29,516	987.8	30.9
Missouri	1,913	29.7	58,107	961.2	30.4
Montana	416	37.6	12,289	1,211.1	29.5
Nebraska	460	23.3	12,899	690.0	28.0
Nevada	1,051	35.1	30,229	1,080.1	28.8
New Hampshire	421	27.5^†^	11,389	860.1	27.1
New Jersey	2,016	20.9	59,604	669.4	29.6
New Mexico	1,145	53.1	35,087	1,683.5	30.6
New York	4,473	20.8	124,315	631.9	27.8
North Carolina	2,876	27.2	85,199	865.4	29.6
North Dakota	216	28.7^†^	6,402	887.1	29.6
Ohio	3,674	29.2	106,752	922.2	29.1
Oklahoma	1,497	37.2	44,920	1,166.8	30.0
Oregon	1,508	33.8	39,705	1,007.9	26.3
Pennsylvania	3,843	27.2	111,516	872.6	29.0
Rhode Island	339	28.8^†^	9,346	887.0	27.6
South Carolina	1,679	32.4	50,141	1,049.5	29.9
South Dakota	283	32.9^†^	8,681	1,029.5	30.7
Tennessee	2,151	30.8	64,392	990.7	29.9
Texas	7,245	27.4	219,901	828.6	30.4
Utah	686	26.2	21,937	755.6	32.0
Vermont	203	27.2^†^	5,085	811.5	25.0
Virginia	2,011	22.7	58,540	709.0	29.1
Washington	2,214	29.1	60,508	866.2	27.3
West Virginia	738	36.1	22,087	1,193.0	29.9
Wisconsin	1,737	27.5	48,122	838.1	27.7
Wyoming	237	38.8^†^	7,329	1,264.3	30.9

* In the Alcohol-Related Disease Impact application (https://www.cdc.gov/ARDI), deaths attributable to excessive alcohol use include deaths from 1) conditions that are 100% alcohol-attributable, 2) deaths caused by acute conditions that involved binge drinking, and 3) deaths caused by chronic conditions that involved medium (>1 to ≤2 drinks of alcohol [women] or >2 to ≤4 drinks [men]) or high (>2 drinks of alcohol [women] or >4 drinks [men]) levels of average daily alcohol consumption.

^†^ The estimate might be unreliable because of suppressed estimates of the number of alcohol-attributable deaths in two or more age groups, and estimates might not account for the total number of alcohol-attributable deaths in the state.

**FIGURE:**
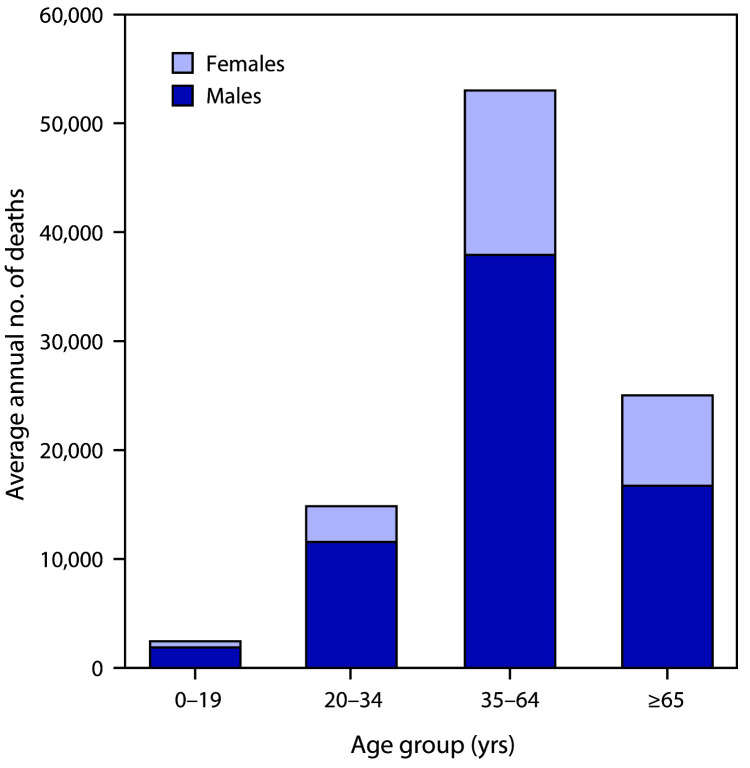
Average annual number of deaths attributable to excessive alcohol use,* by sex and age group — United States, 2011–2015 * In the Alcohol-Related Disease Impact application (https://www.cdc.gov/ARDI), deaths attributable to excessive alcohol use include deaths from 1) conditions that are 100% alcohol-attributable, 2) deaths caused by acute conditions that involved binge drinking, and 3) deaths caused by chronic conditions that involved medium (>1 to ≤2 drinks of alcohol [women] or >2 to ≤4 drinks [men]) or high (>2 drinks of alcohol [women] or >4 drinks [men]) levels of average daily alcohol consumption.

Alcoholic liver disease was the leading chronic cause of alcohol-attributable deaths overall (18,164) and among males (12,887) and females (5,277) (Table 1). Poisonings that involved another substance in addition to alcohol (e.g., drug overdoses) were the leading acute cause of alcohol-attributable deaths overall (11,839) and among females (4,315); suicide associated with excessive alcohol use was the leading acute cause of alcohol-attributable deaths among males (7,711). Conditions wholly attributable to alcohol accounted for 29,068 (30.5%) of all alcohol-attributable deaths and 762,241 (27.6%) of all YPLL.

The national average annual age-adjusted alcohol-attributable death rate was 28.0 per 100,000, and the YPLL per 100,000 was 873.0 (Table 2). The average annual number of alcohol-attributable deaths and YPLL varied across states, ranging from 203 alcohol-attributable deaths in Vermont to 11,026 in California, and from 5,085 YPLL in Vermont to 308,831 in California. Age-adjusted alcohol-attributable death rates among the 40 states with reliable estimates (excluding those with suppressed data where estimates might not account for all the alcohol-attributable deaths in the state) ranged from 20.8 per 100,000 in New York to 53.1 in New Mexico. YPLL per 100,000 ranged from 631.9 in New York to 1,683.5 in New Mexico.

## Discussion

Excessive alcohol use was responsible for approximately 95,000 deaths and 2.8 million YPLL annually in the United States during 2011–2015. This means that an average of 261 Americans die from excessive drinking every day, shortening their lives by an average of 29 years. The majority of these alcohol-attributable deaths involved males, and approximately four in five deaths involved adults aged ≥35 years. The number of alcohol-attributable deaths among adults aged ≥65 years was nearly double that among adults aged 20–34 years. Approximately one half of alcohol-attributable deaths were caused by chronic conditions, but acute alcohol-attributable deaths, all of which were caused by binge drinking, accounted for the majority of the YPLL from excessive drinking.

Little progress has been made in preventing deaths caused by excessive drinking; the average annual estimates of alcohol-attributable deaths and YPLL in this report are slightly higher than estimates for 2006–2010, and the age-adjusted alcohol-attributable death rates are similar (*5*), suggesting that excessive drinking remains a leading preventable cause of death and disability (*1*). From 2006–2010 (*5*) to 2011–2015, average annual deaths caused by alcohol dependence increased 14.2%, from 3,728 to 4,258, and deaths caused by alcoholic liver disease increased 23.6%, from 14,695 to 18,164. These findings are consistent with reported increasing trends in alcohol-induced deaths (e.g., deaths from conditions wholly attributable to alcohol) among adults aged ≥25 years,^†††^ including alcoholic liver disease,^§§§^ as well as with increases in per capita alcohol consumption during the past 2 decades.^¶¶¶^

Age-adjusted alcohol-attributable death rates varied approximately twofold across states, but deaths caused by excessive drinking were common across the country. The differences in alcohol-attributable death and YPLL rates in states might be partially explained by varying patterns of excessive alcohol use, particularly binge drinking, which is affected by state-level alcohol pricing and availability strategies (*6*) and differential access to medical care.

The findings in this report are subject to at least five limitations. First, the prevalence of alcohol consumption ascertained through the Behavioral Risk Factor Surveillance System is based on self-reported data, which substantially underestimates alcohol consumption (*7*). Second, these estimates are conservative, because former drinkers, some of whom might have died from alcohol-related conditions, are not included in the estimates of alcohol-attributable deaths and YPLL for partially alcohol-attributable causes of death. Third, direct alcohol-attributable fraction estimates for some chronic and acute conditions rely on data older than that of 2011–2015 (*4*) and might not accurately represent the proportion of excessive drinkers among persons who died of some conditions (e.g., drug overdoses) during that period. This emphasizes the importance of more timely information on alcohol involvement and various health conditions. Fourth, several conditions partially related to alcohol (e.g., tuberculosis, human immunodeficiency virus, and acquired immunodeficiency syndrome)**** are not included because published risk estimates were not available. Finally, the alcohol-attributable deaths and YPLL are based on alcohol-related conditions that were listed as the underlying (i.e., primary) cause of death, and not as a multiple cause of death, yielding conservative estimates.

The implementation of effective population-based strategies for preventing excessive drinking, such as those recommended by the Community Preventive Services Task Force (e.g., increasing alcohol taxes and regulating the number and concentration of alcohol outlets), could reduce alcohol-attributable deaths and YPLL. These strategies can complement other population-based prevention strategies that focus on health risk behaviors associated with excessive alcohol use, such as safer prescribing practices to reduce opioid misuse and overdoses (*8*,*9*) and alcohol-impaired driving interventions (*10*).

**Summary**

**What is already known about this topic?**

Excessive drinking is a leading cause of preventable death in the United States and is associated with numerous health and social problems.

**What is added by this report?**

During 2011–2015, excessive drinking was responsible for an average of 95,158 deaths (261 per day) and 2.8 million years of potential life lost (29 years lost per death, on average) in the United States each year.

**What are the implications for public health practice?**

Widespread implementation of prevention strategies, including those recommended by the Community Preventive Services Task Force (e.g., increasing alcohol taxes and regulating the number and concentration of places that sell alcohol) could help reduce deaths and years of potential life lost from excessive drinking.

## Footnotes

* https://www.cdc.gov/ARDI.

† https://www.thecommunityguide.org/topic/excessive-alcohol-consumption.

§ https://www.cdc.gov/alcohol/ardi/alcohol-related-icd-codes.html.

¶ https://www.cdc.gov/alcohol/ardi/methods.html.

** https://www.cdc.gov/brfss/.

†† https://www-fars.nhtsa.dot.gov/Crashes/CrashesAlcohol.aspx.

§§ Conditions that that are 100% alcohol-attributable include 13 chronic conditions (alcoholic psychosis, alcohol abuse, alcohol dependence syndrome, alcohol polyneuropathy, degeneration of the nervous system caused by alcohol use, alcoholic myopathy, alcohol cardiomyopathy, alcoholic gastritis, alcoholic liver disease, alcohol-induced acute pancreatitis, alcohol-induced chronic pancreatitis, fetal alcohol syndrome, and fetus and newborn affected by maternal use of alcohol) and two acute conditions (suicide by and exposure to alcohol and alcohol poisoning).

¶¶ https://www.cdc.gov/mmwr/preview/mmwrhtml/00001773.htm.

*** https://www.cdc.gov/nchs/data/statnt/statnt20.pdf.

††† https://www.cdc.gov/mmwr/volumes/68/wr/mm6833a5.htm.

§§§ https://pubs.niaaa.nih.gov/publications/surveillance111/Cirr15.htm.

¶¶¶ https://pubs.niaaa.nih.gov/publications/surveillance110/CONS16.htm.

**** https://apps.who.int/iris/bitstream/handle/10665/274603/9789241565639-eng.pdf?ua.
